# Review of the mesh-web spider genus *Sudesna* Lehtinen, 1967 (Araneae, Dictynidae) from Xizang, China

**DOI:** 10.3897/zookeys.1278.187037

**Published:** 2026-04-30

**Authors:** Lu-Yu Wang, Zhi-Sheng Zhang, Yong-Qiang Xu

**Affiliations:** 1 Key Laboratory of Eco-environments in Three Gorges Reservoir Region (Ministry of Education), School of Life Sciences, Southwest University, Chongqing 400715, China School of Life Sciences, Southwest University Chongqing China; 2 Institute of Plateau Biology of Xizang Autonomous Region, Lhasa 850001, Xizang Autonomous Region, China Institute of Plateau Biology of Xizang Autonomous Region Lhasa China; 3 Medog Biodiversity Observation and Research Station of Xizang Autonomous Region, Medog, China Medog Biodiversity Observation and Research Station of Xizang Autonomous Region Medog China

**Keywords:** Description, morphology, new combination, new species, taxonomy

## Abstract

The genus *Sudesna* Lehtinen, 1967 (Araneae, Dictynidae) from Xizang, China is reviewed, including one new combination and four new species: *S. linzhiensis* (Hu, 2001), **comb. nov**., *S. chayu***sp. nov**. (♂♀), *S. mii***sp. nov**. (♂♀), *S. medog***sp. nov**. (♂) and *S. wangi***sp. nov**. (♂♀). Additionally, the males of *S. flavipes* (Hu, 2001) and *S. linzhiensis* (Hu, 2001) are described here for the first time.

## Introduction

The genus *Sudesna* Lehtinen, 1967, is a small genus within the family Dictynidae O. Pickard-Cambridge, 1871, comprising 13 recognized species worldwide, distributed across Australia and South, Southeast, and East Asia (WSC 2026; Wang et al. 2025). Among these, the majority (9) are found exclusively in China. The type species, *S. hedini* (Schenkel, 1936), occurs in China and South Korea. Other notable species include *S. anaulax* (Simon, 1908) from Australia, *S. grammica* (Simon, 1893) from the Philippines, and *S. grossa* (Simon, 1906) from India (WSC 2026). This highlights China as the region with the greatest diversity of *Sudesna* species.

Xizang, the second-largest provincial-level administrative division in China, is not only a hotspot for studying biological evolution but also an area where spider diversity remains poorly understood (Wang et al. 2024). Currently, only eight species from six genera of cribellate spiders have been recorded in Xizang, with just one belonging to *Sudesna*: *S. flavipes* (Hu, 2001) (Hu 2001).

During our examination of *Sudesna* specimens collected from Xizang, we identified four new species: *S. chayu* sp. nov., *S. mii* sp. nov., *S. medog* sp. nov. and *S. wangi* sp. nov. Additionally, we describe the males of *S. flavipes* (Hu, 2001) and *Dictyna linzhiensis* Hu, 2001 for the first time. The latter species had previously been treated as *incertae sedis* (Esyunin and Sozontov 2016). Based on examination of both male and female specimens, we found that the palpal and epigynal structures align with the diagnostic characteristics of the genus *Sudesna*. Consequently, we hereby transfer *D. linzhiensis* to the genus *Sudesna* and propose the new combination: *S. linzhiensis* (Hu, 2001), comb. nov.

## Materials and methods

All specimens are preserved in 75% ethanol and examined, illustrated, photographed, and measured using a Leica M205A stereomicroscope equipped with a drawing tube, a Leica DFC450 Camera, and LAS v. 4.6 software. Male palps and epigynes were examined and illustrated after dissection. Epigynes were cleared by immersing them in a pancreatin solution (Álvarez-Padilla and Hormiga 2007). Eye sizes were measured as the maximum dorsal diameter. Leg measurements are shown as: total length (femur, patella and tibia, metatarsus, tarsus). All measurements are in millimetres. Specimens examined here are deposited in the Collection of Spiders, School of Life Sciences, Southwest University, Chongqing, China (SWUC).

Abbreviations used in the text: **AA**, anterior arm of conductor; **ALE**, anterior lateral eye; **AME**, anterior median eye; **CD**, copulatory duct; **CO**, copulatory opening; **Ct**, ctenidia; **Em**, embolus; **FD**, fertilization duct; **MOA**, median ocular area; **PA**, posterior arm of conductor; **PLE**, posterior lateral eye; **PME**, posterior median eye; **RTA**, retrolateral tibial apophysis; **S**, spermathecal; **SH**, spermathecal head.

## Taxonomy

### Family Dictynidae O. Pickard-Cambridge, 1871 (卷叶蛛科)

**Subfamily Dictyninae O. Pickard-Cambridge, 1871 (**卷叶蛛亚科)

#### Genus Sudesna

Taxon classificationAnimaliaAraneaeDictynidae

Lehtinen, 1967

DE000620-8FAE-5451-9EA8-87B2DA8D6365

##### Chinese name.

苏蛛属.

##### Diagnosis.

See Wang, Peng and Zhang (2025).

##### Description.

Diminutive in size (1.60–4.57). Dorsum of prosoma pale, darker to brown, with high cephalic area. Fovea absent. Cervical groove distinct, radial furrows indistinct. Eight eyes in 2 rows; eyes located on eye tubercles; eye tubercles coloured same as carapace. Chelicerae stout, yellowish to brown, with small yellow lateral condyles, 3–4 promarginal and 1–3 retromarginal teeth. Endites yellow, longer than wide. Labium yellow-brown, as long as wide. Sternum yellow-brown, with truncated anterior margin and blunt posterior margin. Legs yellowish to brown, patella with a small protrusion. Leg formula: 1243, 1432 or 1423. Opisthosoma oval. Dorsum pale to darker brown, with some small, white, scale-like markings near midline. Venter of abdomen yellow-brown, with small, undivided cribellum. Spinnerets short and yellowish brown (Wang et al. 2025).

##### Remarks.

Species of *Sudesna* are indeed very similar to each other, making morphological identification challenging. However, the posterior arm of the conductor, the spermathecae, and the spermathecal heads are strongly sclerotized and exhibit stable, species-specific structures, making them reliable diagnostic features for species delimitation within the genus.

#### Sudesna chayu sp. nov.

Taxon classificationAnimaliaAraneaeDictynidae

F7F68A75-D716-508E-888E-8299851F65B4

https://zoobank.org/37BABFF8-F423-4F5C-814D-EBE93F48C5FE

Figs 1, 2, 13

##### Chinese name.

察隅苏蛛.

##### Type material.

***Holotype***: ♂ (SWUC-T-DI-20-01), • China, Xizang, Nyingchi City, Chayu County, No. 201 road, 64 km, 29°19'41"N, 97°08'08"E, elev. 3903 m, 25 June 2018, L.Y. Wang, Z.S. Wu and Y.N. Mu leg. ***Paratypes***: • 2 ♂ 3 ♀ (SWUC-T-DI-20-02 to 06), with same data as for holotype.

##### Etymology.

The specific name is derived from the county where the type locality is located; used as a noun in apposition.

##### Diagnosis.

**Figure 1.**
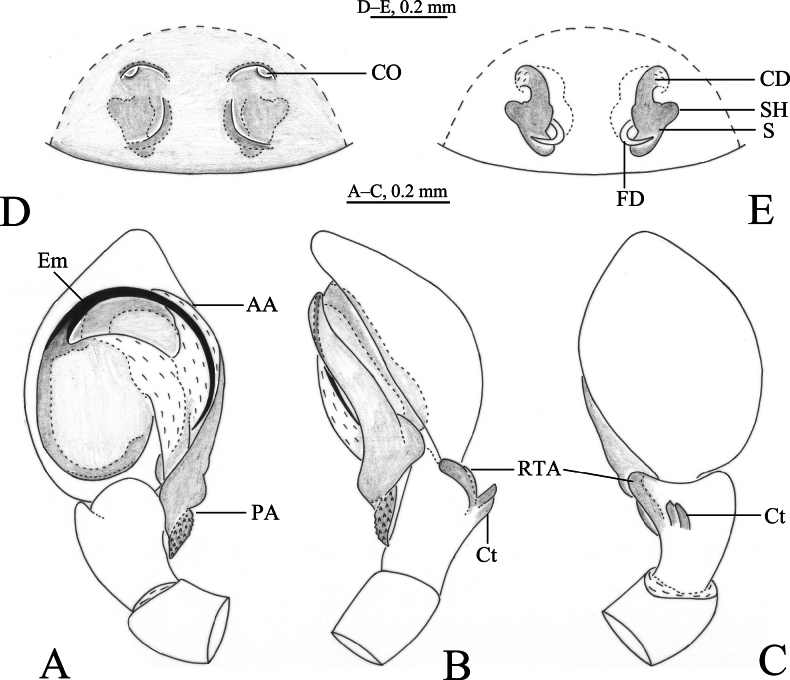
*Sudesna chayu* sp. nov. holotype male (**A–C**) and paratype female (**D, E**). **A**. Left male palp, ventral view; **B**. Same, retrolateral view; **C**. Same, dorsal view; **D**. Epigyne, ventral view; **E**. Vulva, dorsal view.

**Figure 2.**
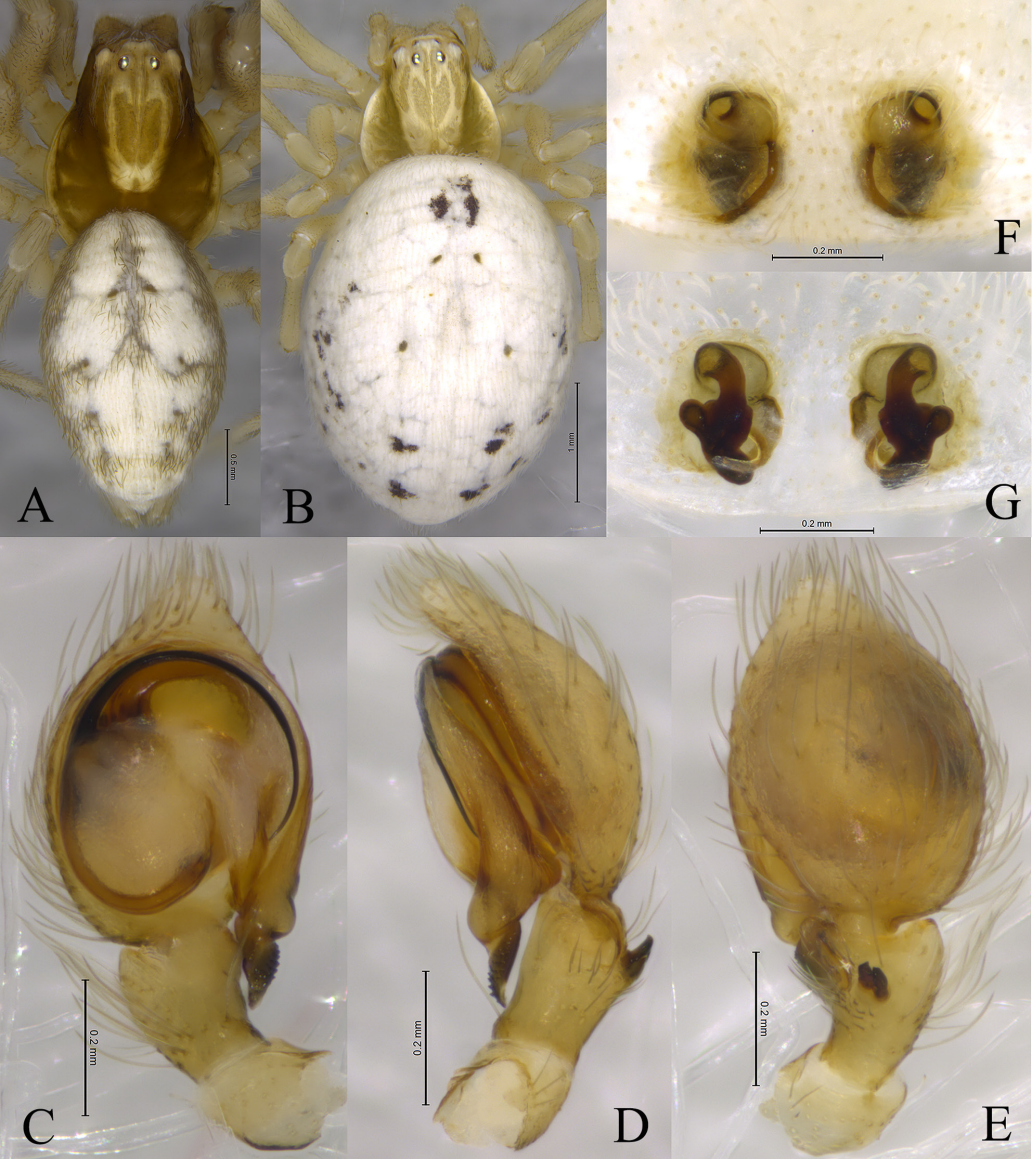
*Sudesna chayu* sp. nov. holotype male (**A, C–E**) and paratype female (**B, F, G**). **A**. Male habitus, dorsal view; **B**. Female habitus, dorsal view; **C**. Left male palp, ventral view; **D**. Same, retrolateral view; **E**. Same, dorsal view; **F**. Epigyne, ventral view; **G**. Vulva, dorsal view.

This new species is similar to *S. mii* sp. nov. (Figs 7, 8) in having a semicircular embolus and short copulatory ducts, but it can be distinguished by the rounded end of the dorsal edge of the conductor (vs slightly pointed) (Figs 1B, 2D), the semicircular copulatory openings (vs 6-shaped) (Figs 1D, 2F), and the indistinct stalk of spermathecal heads (vs distinct) (Figs 1E, 2G).

##### Description.

**Male holotype** (Fig. 2A) total length 2.67. Prosoma 1.08 long, 0.97 wide; Opisthosoma 1.62 long, 1.04 wide. Eye sizes and interdistances: AME 0.04, ALE 0.08, PME 0.07, PLE, 0.07; AME–AME 0.11, AME–ALE 0.08, PME–PME 0.10, PME–PLE 0.11, ALE–PLE 0.03. MOA 0.19 long, anterior width 0.19, posterior width 0.22. Clypeus height 0.11. Chelicerae stout, brown, with 3 promarginal teeth and 2 retromarginal teeth. Legs yellowish. Leg measurements: I 3.48 (1.06, 1.23, 0.73, 0.46); II 3.31 (1.05, 1.15, 0.66, 0.45); III 2.54 (0.78, 0.82, 0.59, 0.35); IV 2.89 (0.86, 1.00, 0.69, 0.34).

***Palp*** (Figs 1A–C, 2C–E). Tibia with 2 ctenidia dorsally, located at 1/3 length of tibia from distal-most part. Retrolateral tibial apophysis triangular in dorsal view. Embolus semicircular and originating at about 10:00 o’clock position and terminating at about 4:00 o’clock position. Anterior arm of conductor (AA) short and membranous; posterior arm (PA) slightly twisted with a pointed, scaly tip.

**Female paratype** (SWUC-T-DI-20-02, Fig. 2B) total length 3.35. Prosoma 1.01 long, 0.98 wide; opisthosoma 2.55 long, 1.85 wide. Eye sizes and interdistances: AME 0.05, ALE 0.07, PME 0.07, PLE, 0.07; AME–AME 0.11, AME–ALE 0.08, PME–PME 0.11, PME–PLE 0.11, ALE–PLE 0.04. MOA 0.18 long, anterior width 0.19, posterior width 0.24. Clypeus height 0.09. Legs yellowish. Leg measurements: I 3.11 (0.99, 1.01, 0.66, 0.45); II 3.09 (0.97, 1.01, 0.64, 0.47); III 2.58 (0.84, 0.79, 0.57, 0.38); IV 3.07 (0.97, 1.11, 0.67, 0.32).

***Epigyne*** (Figs 1D, 1E, 2F, 2G). Copulatory openings semicircular, facing away from each other, separated by about 6 times their width. Copulatory ducts slightly sclerotized, short, slightly curved. Spermatheca curved, spermathecal heads somewhat ball-shaped. Fertilization ducts length about 2.5 times the width of copulatory openings, curved as semicircular, directed laterally.

##### Variation.

Male (*N* = 3) total length 2.44–2.67, female (*N* = 3) total length 2.47–3.35

##### Distribution.

Known only from the type locality, Xizang, China (Fig. 13).

#### Sudesna flavipes

Taxon classificationAnimaliaAraneaeDictynidae

(Hu, 2001)

FA7A22D3-0DDB-5E7C-913E-2DAAA13878B6

Figs 3, 4, 13

Dictyna flavipes Hu, 2001: 99, figs 19.1–2 (♀).
Sudesna flavipes : Esyunin and Sozontov 2016: 202.

##### Chinese name.

黄足苏蛛.

##### Material examined.

**China**, **Xizang**: • 24 ♂ 4 ♀, Nyingchi City, Bayi Town, 29°35'44"N, 94°25'54"E, elev. 2972 m, 2.05.2002, M.S. Zhu leg. • 10 ♂ 6 ♀, Nyingchi City, Bayi Town, Baishuwang, 29°37'01"N, 94°24'12"E, elev. 2992 m, 17.08.2002, M.S. Zhu leg. • 10 ♂ 11 ♀, Nyingchi City, Bayi Town, Baishuwang, 18.08.2009, Z.Z. Yang, Z.X. Li, L.Y. Wang leg. • 104 ♂ 51 ♀, Nyingchi City, Mainling County, 29°13'03"N, 94°12'55"E, elev. 2914 m, 19.08.2002, M.S. Zhu leg. • 1 ♂, Shannan City, Nedong District, Zedang Town, 29°13'56"N, 91°46'36"E, elev. 3616 m, 25.08.2002, M.S. Zhu leg.

##### Diagnosis.

**Figure 3.**
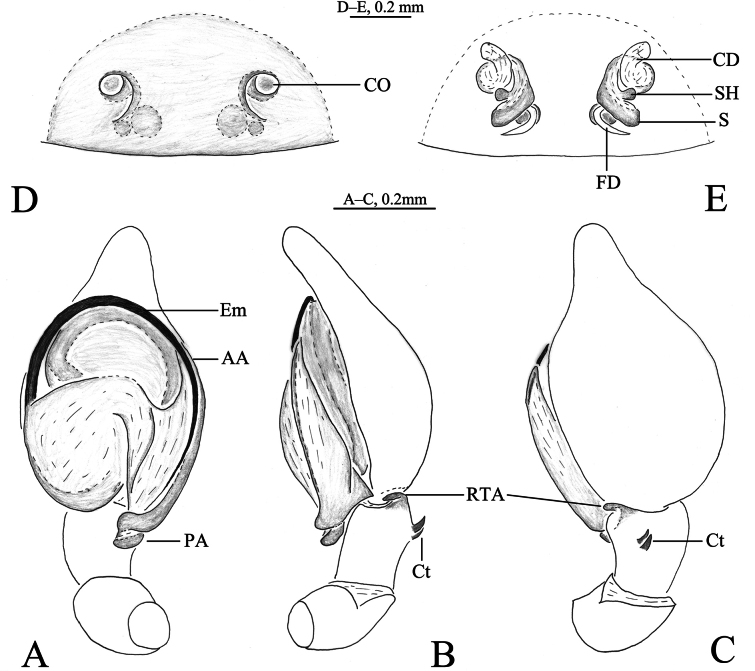
*Sudesna flavipes* (Hu, 2001). **A**. Left male palp, ventral view; **B**. Same, retrolateral view; **C**. Same, dorsal view; **D**. Epigyne, ventral view; **E**. Vulva, dorsal view.

**Figure 4.**
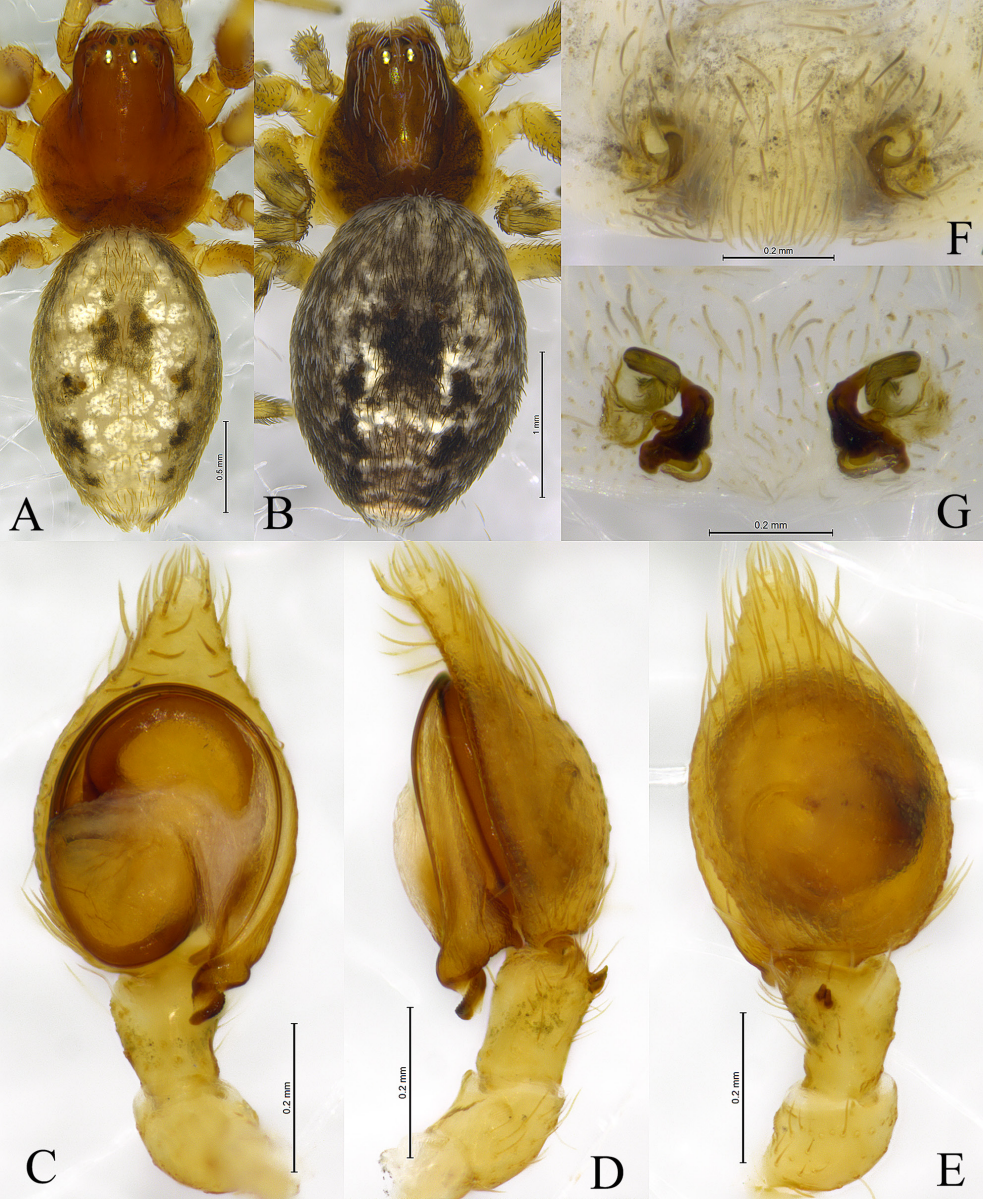
*Sudesna flavipes* (Hu, 2001). **A**. Male habitus, dorsal view; **B**. Female habitus, dorsal view; **C**. Left male palp, ventral view; **D**. Same, retrolateral view; **E**. Same, dorsal view; **F**. Epigyne, ventral view; **G**. Vulva, dorsal view.

The male of this species is similar to *S. wangi* sp. nov. (Figs 11A–C, 12C–E) in having somewhat O-shaped embolus, and spiral, with a rounded end posterior arm (PA) of the conductor, but it can be distinguished by the embolus originating at 9:00 o’clock position (vs 10:00 o’clock position), the slender posterior arm of conductor (vs fat) (Figs 3A, 3B, 4C, 4D). The female of this species can be distinguished from all other congeners by possessing long copulatory ducts with two turns.

##### Description.

**Male** (Fig. 4A) total length 2.59. Prosoma 1.14 long, 0.99 wide; Opisthosoma 1.55 long, 1.07 wide. Eye sizes and interdistances: AME 0.06, ALE 0.07, PME 0.07, PLE, 0.08; AME–AME 0.06, AME–ALE 0.05, PME–PME 0.09, PME–PLE 0.09, ALE–PLE 0.02. MOA 0.18 long, anterior width 0.20, posterior width 0.21. Clypeus height 0.10. Chelicerae stout, brown, with 3 promarginal teeth and 1 retromarginal tooth. Legs yellowish, with black pigmentation. Leg measurements: I 4.27 (1.31, 1.52, 0.88, 0.56); II 3.64 (1.14, 1.24, 0.76, 0.50); III 2.79 (0.88, 0.92, 0.61, 0.38); IV 3.10 (0.95, 1.05, 0.72, 0.38). Leg formula: 1243.

***Palp*** (Figs 3A–C, 4C–E). Tibia with 2 ctenidia dorsally, located at 1/3 length of tibia from distal-most part. Retrolateral tibial apophysis hook-shaped in retrolateral view. Embolus somewhat O-shaped, originating at about the 9:00 o’clock position and terminating at about the 5:30 o’clock position. Anterior arm of conductor (AA) short and membranous; posterior arm (PA) spiral, with rounded tip.

**Female** (Fig. 4B) total length 3.16. Prosoma 1.25 long, 1.15 wide; opisthosoma 2.20 long, 1.49 wide. Eye sizes and interdistances: AME 0.06, ALE 0.07, PME 0.07, PLE, 0.08; AME–AME 0.11, AME–ALE 0.07, PME–PME 0.10, PME–PLE 0.12, ALE–PLE 0.03. MOA 0.20 long, anterior width 0.20, posterior width 0.22. Clypeus height 0.10. Legs yellowish, with black pigmentation. Leg measurements: I 4.14 (1.31, 1.45, 0.87, 0.51); II 3.72 (1.18, 1.27, 0.78, 0.49); III 3.07 (1.00, 1.02, 0.67, 0.38); IV 3.60 (1.17, 1.25, 0.79, 0.39). Leg formula: 1243.

***Epigyne*** (Figs 3D, 3E, 4F, 4G). Copulatory openings semicircular, facing away from each other, separated about 7.5 times their width. Copulatory ducts membranous, long, with two turns. Spermatheca peanut-shaped, spermathecal heads somewhat ball-shaped. Fertilization ducts length about 2.5 times the width of copulatory openings, curved as semicircular, directed laterally.

##### Distribution.

China (Xizang) (Fig. 13).

#### Sudesna linzhiensis

Taxon classificationAnimaliaAraneaeDictynidae

(Hu, 2001) comb. nov.

8313DC89-5466-57AA-94AC-345E79F9D46F

Figs 5, 6, 13

Dictyna linzhiensis Hu, 2001: 102, figs 22.1–5 (♀)
“Dictyna” *linzhiensis*: Esyunin and Sozontov 2016: 204

##### Chinese name.

林芝苏蛛.

##### Material examined.

**China, Xizang**: • 1 ♂ 15 ♀, Nyingchi City, Sejila Mt., 29°37'06"N, 94°42'12"E, elev. 4179 m, 17.08.2009, Z.Z. Yang, Z.X. Li, L.Y. Wang leg. • 3 ♀, Nyingchi City, Chayu County, Guyu Town, 29°05'32"N, 97°17'13"E, elev.3196 m, 25.06.2018, L.Y. Wang, Z.S. Wu, Y.N. Mu leg. • 4 ♀, Qamdo City, Riwoqe County, Yiri Township, 31°33'49"N, 96°30'14"E, elev. 4478 m, 3.08.2017, T. Lu leg. • 1 ♀, Riwoqe County, Riwoqe Town, 31°26'29"N, 96°23'57"E, elev. 4035 m, 22.05.2017, T. Lu leg. • 8 ♀, Riwoqe County, Binda Township, 31°07'54"N, 96°39'31"E, elev. 3707 m, 21.06.2018, L.Y. Wang, Z.S. Wu and Y.N. Mu leg. • 1 ♀, Markam County, 29°41'48"N, 98°35'37"E, elev. 3878 m, 16.05.2019, L.Y. Wang leg. • 2 ♀, Markam County, Rongme Town, Lawu Village, 29°43'03"N, 98°25'58"E, elev. 3563 m, 16.05.2019, L.Y. Wang leg.

##### Diagnosis.

**Figure 5.**
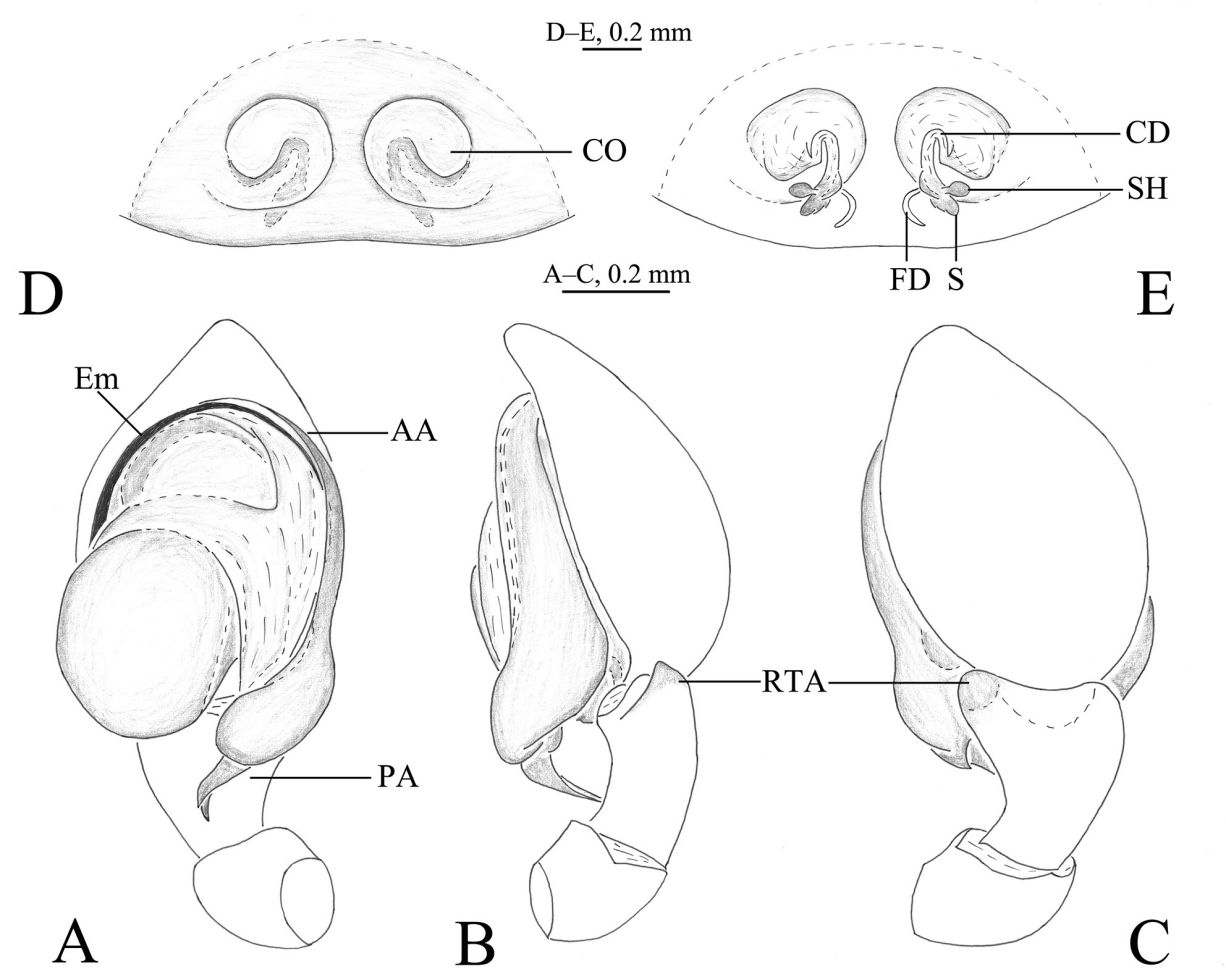
*Sudesna linzhiensis* (Hu, 2001). **A**. Left male palp, ventral view; **B**. Same, retrolateral view; **C**. Same, dorsal view; **D**. Epigyne, ventral view; **E**. Vulva, dorsal view.

**Figure 6.**
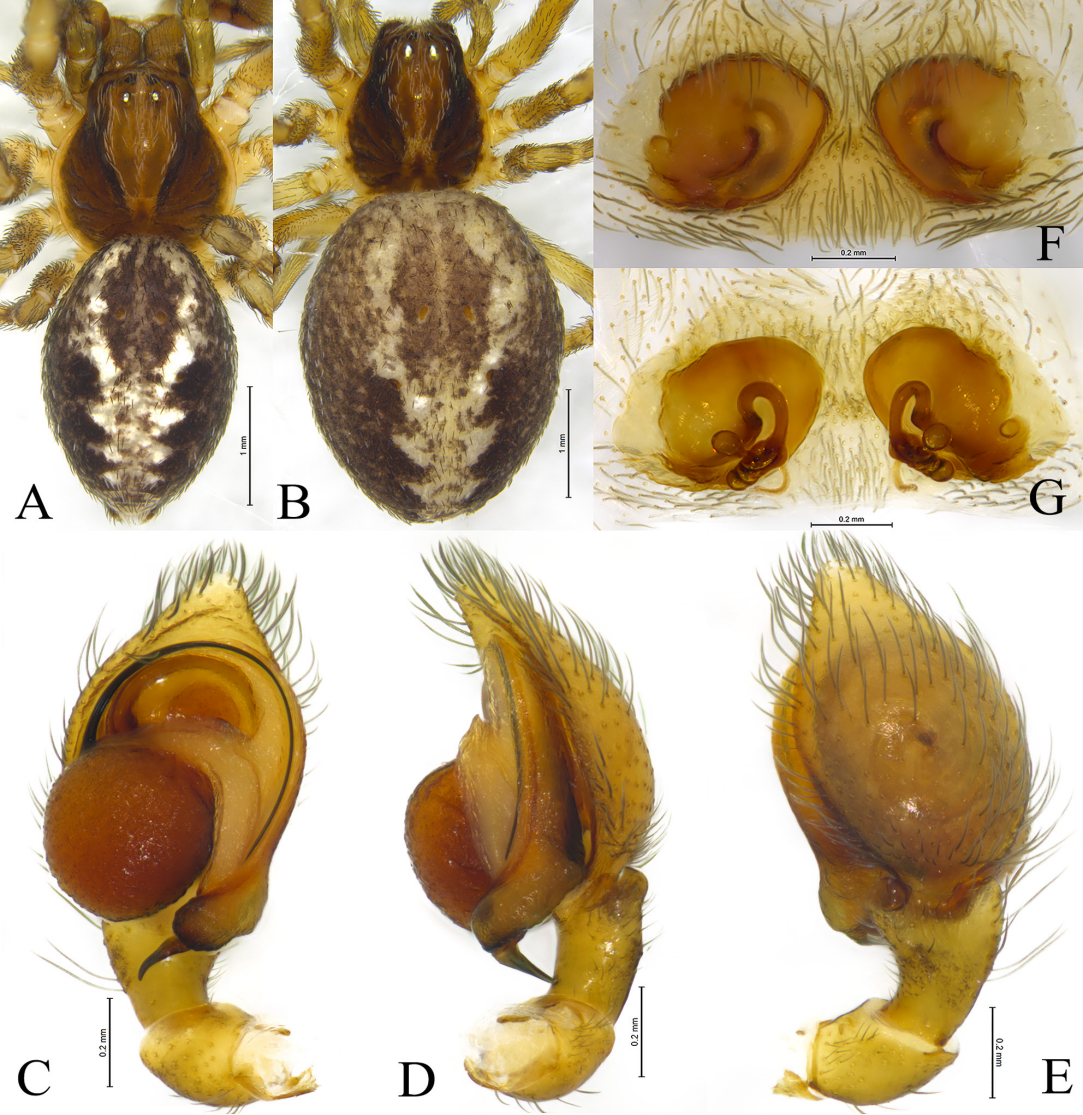
*Sudesna linzhiensis* (Hu, 2001). **A**. Male habitus, dorsal view; **B**. Female habitus, dorsal view; **C**. Left male palp, ventral view; **D**. Same, retrolateral view; **E**. Same, dorsal view; **F**. Epigyne, ventral view; **G**. Vulva, dorsal view.

The male of this species is similar to *S. yangi* Wang, Peng & Zhang (Wang et al. 2025: 144, figs 13A–C, 14C–E) in having a long and sharp posterior arm (PA) of the conductor, and both of them without ctenidia on the tibia, but it can be distinguished by the triangular (retrolateral view) retrolateral tibial apophysis (vs hook-shaped) (Figs 5B, 6D), the inflated embolus base (vs flat) (Figs 5A, 5B, 6C, 6D). The female of this species is distinguished from all other congeners by possessing a large atrium.

##### Description.

**Male** (Fig. 6A) total length 3.22. Prosoma 1.27 long, 1.12 wide; Opisthosoma 2.05 long, 1.43 wide. Eye sizes and interdistances: AME 0.07, ALE 0.07, PME 0.06, PLE, 0.07; AME–AME 0.06, AME–ALE 0.09, PME–PME 0.12, PME–PLE 0.12, ALE–PLE 0.03. MOA 0.20 long, anterior width 0.21, posterior width 0.25. Clypeus height 0.10. Chelicerae stout, brown, with 3 promarginal teeth and 1 retromarginal tooth. Legs yellowish, with brown pigmentation. Leg measurements: I 3.86 (1.15, 1.43, 0.80, 0.48); II 3.50 (1.10, 1.22, 0.72, 0.46); III 2.95 (0.95, 0.97, 0.63, 0.40); IV 3.40 (1.01, 1.23, 0.76, 0.40).

***Palp*** (Figs 5A–C, 6C–E). Tibia without ctenidia. Retrolateral tibial apophysis triangular in retrolateral view. Embolus semicircular and originating at about the 9:00 o’clock position and terminating at about the 4:30 o’clock position. Anterior arm of conductor (AA) short and membranous; posterior arm (PA) spiral, with a pointed tip.

**Female** (Fig. 6B) total length 3.35. Prosoma 1.34 long, 1.13 wide; opisthosoma 2.25 long, 1.63 wide. Eye sizes and interdistances: AME 0.06, ALE 0.08, PME 0.06, PLE, 0.07; AME–AME 0.06, AME–ALE 0.06, PME–PME 0.11, PME–PLE 0.11, ALE–PLE 0.03. MOA 0.18 long, anterior width 0.20, posterior width 0.22. Clypeus height 0.10. Legs yellowish, with brown pigmentation. Leg measurements: I 3.72 (1.13. 1.34. 0.75. 0.50); II 3.42 (1.06. 1.20. 0.69. 0.47); III 2.96 (0.93. 1.00. 0.62. 0.41); IV 3.48 (1.05. 1.25. 0.77. 0.41).

***Epigyne*** (Figs 5D, 5E, 6F, 6G). Copulatory openings semicircular, facing away from each other, separated about 2.7 times their width. Copulatory ducts slightly sclerotized, almost n-shaped. Spermatheca peanut-shaped, spermathecal heads ball-shaped. Fertilization ducts semicircular, directed laterally, as long as the width of copulatory openings.

##### Distribution.

China (Xizang) (Fig. 13).

##### Remarks.

Based on the diagnostic characteristics of the genus *Dictyna*, the male palpal tibia possesses well-developed ctenidia, the copulatory openings are indistinct, and the copulatory ducts large and sac-like (Marusik 2022). However, in *D. linzhiensis*, the male palpal tibia lacks ctenidia, the copulatory openings are large, distinct, and anteriorly located, and the copulatory ducts are tube-like, all of which are consistent with the diagnostic features of *Sudesna* (Wang et al. 2025). Accordingly, we transfer *D. linzhiensis* to *Sudesna* as *S. linzhiensis*, new combination.

#### Sudesna mii sp. nov.

Taxon classificationAnimaliaAraneaeDictynidae

AEA2352E-14CE-5323-A1E7-E2EA3677F30B

https://zoobank.org/839F6B36-1C99-436E-A96C-0172B807E663

Figs 7, 8, 13

##### Chinese name.

米氏苏蛛.

##### Type material.

***Holotype***: ♂ (SWUC-T-DI-21-01) • China, Xizang, Mainling City, Pai Town, Dalin village, 29°36'57"N, 94°53'00"E, elev. 3203 m, 12.08.2023, X.Q. Mi leg. ***Paratypes***: • 1 ♂ 2 ♀ (SWUC-T-DI-21-02 to 04), same data as holotype.

##### Etymology.

This species is named after the collector of the type material; a noun in genitive case.

##### Diagnosis.

**Figure 7.**
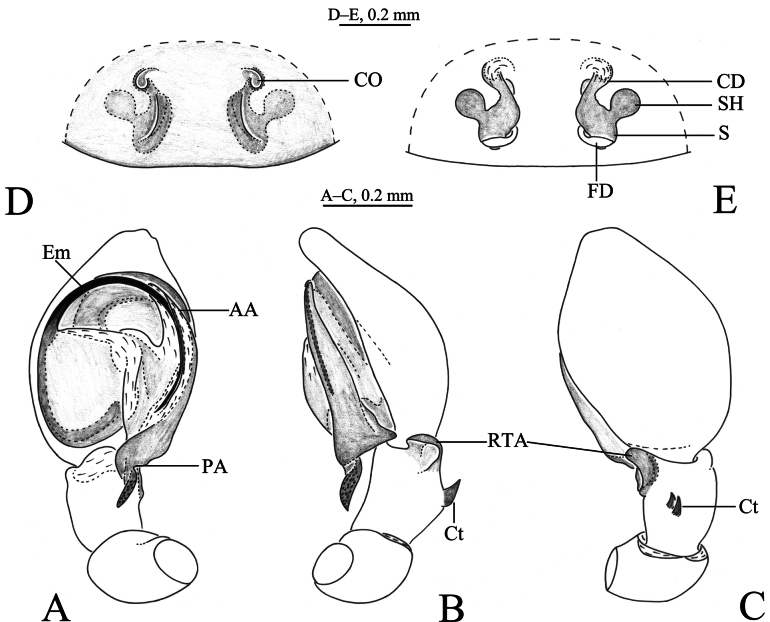
*Sudesna mii* sp. nov. holotype male (**A–C**) and paratype female (**D, E**). **A**. Left male palp, ventral view; **B**. Same, retrolateral view; **C**. Same, dorsal view; **D**. Epigyne, ventral view; **E**. Vulva, dorsal view.

**Figure 8.**
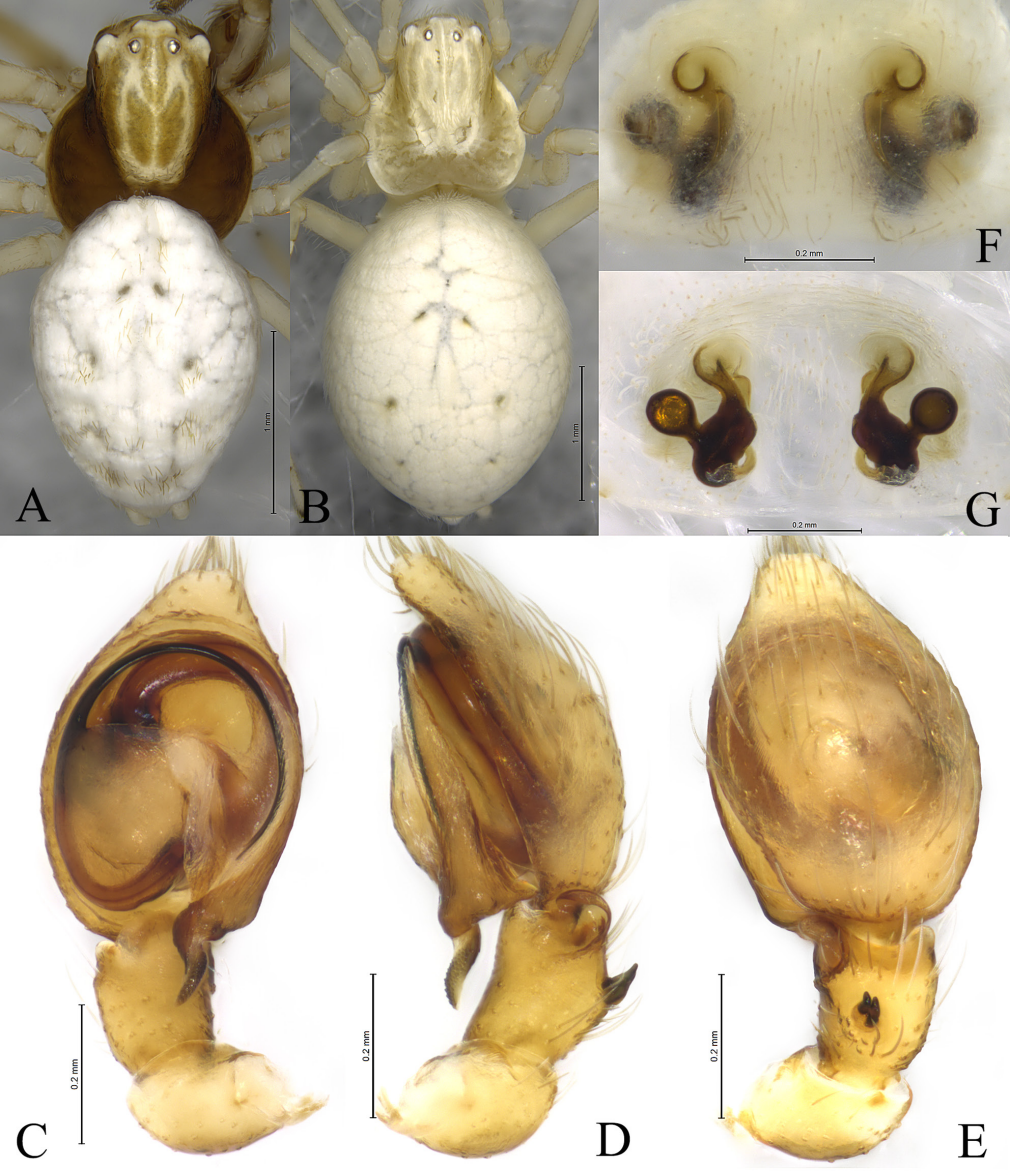
*Sudesna mii* sp. nov. holotype male (**A, C–E**) and paratype female (**B, F, G**). **A**. Male habitus, dorsal view; **B**. Female habitus, dorsal view; **C**. Left male palp, ventral view; **D**. Same, retrolateral view; **E**. Same, dorsal view; **F**. Epigyne, ventral view; **G**. Vulva, dorsal view.

See the diagnosis of *S. chayu* sp. nov. This new species is also similar to *S. hainan* Wang, Peng & Zhang, 2025. (Wang et al. 2025: 136, figs 7A–E, 8A–G) in having a semicircular embolus, somewhat S-shaped posterior arm (PA) of the conductor, short copulatory ducts and large spermathecal heads, but it can be distinguished by the triangular (dorsal view) retrolateral tibial apophysis (vs semicircular, lamellate) (Figs 7C, 8E), the originating at 10:00 o’clock position of embolus (vs 11:30) (Figs 7A, 8C), and the 6-shaped copulatory openings (vs 9-shaped) (Figs 7D, 8F).

##### Description.

**Male holotype** (Fig. 8A) total length 2.70. Prosoma 1.33 long, 1.15 wide; Opisthosoma 1.71 long, 1.28 wide. Eye sizes and interdistances: AME 0.06, ALE 0.08, PME 0.08, PLE, 0.09; AME–AME 0.15, AME–ALE 0.13, PME–PME 0.15, PME–PLE 0.16, ALE–PLE 0.02. MOA 0.23 long, anterior width 0.26, posterior width 0.30. Clypeus height 0.14. Chelicerae stout, yellow-brown, with 3 promarginal teeth and 2 retromarginal teeth. Legs yellowish. Leg measurements: I 3.59 (1.10, 1.26, 0.72, 0.51); II 3.42 (1.03, 1.19, 0.71, 0.49); III 2.59 (0.82, 0.87, 0.57, 0.33); IV 2.98 (0.95, 1.00, 0.69, 0.34).

***Palp*** (Figs 7A–C, 8C–E). Tibia with 2 ctenidia dorsally, located at middle parts. Retrolateral tibial apophysis hood-shaped in retrolateral view and triangular in dorsal view. Embolus semicircular and originating at about 9:30 o’clock position and terminating at about 4:30 o’clock position. Anterior arm of conductor (AA) short and membranous; posterior arm (PA) backwards S-shaped in retrolateral view, with a blunt, scaly tip.

**Female paratype** (SWUC-T-DI-21-02, Fig. 8B) total length 3.78. Prosoma 1.31 long, 1.23 wide; opisthosoma 2.44 long, 1.87 wide. Eye sizes and interdistances: AME 0.05, ALE 0.07, PME 0.07, PLE, 0.06; AME–AME 0.14, AME–ALE 0.12, PME–PME 0.15, PME–PLE 0.16, ALE–PLE 0.02. MOA 0.23 long, anterior width 0.23, posterior width 0.29. Clypeus height 0.11. Leg measurements: I 3.73 (1.16, 1.24, 0.79, 0.54); II 3.59 (1.15, 1.17, 0.77, 0.50); III 3.13 (1.01, 1.05, 0.65, 0.42); IV 3.73 (1.16, 1.32, 0.83, 0.42).

***Epigyne*** (Figs 7D, 7E, 8F, 8G). Copulatory openings 6-shaped, facing each other, separated about 5.7 times their width. Copulatory ducts membranous, two times longer than wide. Spermatheca peanut-shaped, spermathecal heads large, ball-shaped. Fertilization ducts length about 2.5 times the width of copulatory openings, curved as semicircular, directed laterally.

##### Variation.

Male (*N* = 2) total length 2.70–2.94, female (*N* = 2) total length 3.61–3.78

##### Distribution.

Known only from the type locality, Xizang, China (Fig. 13).

#### Sudesna medog sp. nov.

Taxon classificationAnimaliaAraneaeDictynidae

B06D6F8D-9D88-5BAF-85CB-1888CD19B1B2

https://zoobank.org/6AD9285C-42CD-47A1-9F11-E1E2193919D0

Figs 9, 10, 13

##### Chinese name.

墨脱苏蛛.

##### Type material.

***Holotype***: ♂ (SWUC-T-DI-22-01) • China, Xizang, Nyingchi City, Medog County, Beibeng Township, Gelin Village, 29°13'25"N, 95°11'07"E, elev. 1545 m, Q.L. Lu leg.

##### Etymology.

The specific name is derived from the county where the type locality is located; used as a noun in apposition.

##### Diagnosis.

This new species can be distinguished from all other congeners by the large and serrated posterior arm (PA) of the conductor.

##### Description.

**Male holotype** (Fig. 10A) total length 1.99. Prosoma 0.95 long, 0.88 wide; Opisthosoma 1.09 long, 0.72 wide. Eye sizes and interdistances: AME 0.04, ALE 0.05, PME 0.05, PLE, 0.05; AME–AME 0.09, AME–ALE 0.06, PME–PME 0.10, PME–PLE 0.09, ALE–PLE 0.01. MOA 0.13 long, anterior width 0.17, posterior width 0.18. Clypeus height 0.09. Chelicerae stout, brown, with 4 promarginal teeth and 1 retromarginal tooth. Legs yellowish, with black pigmentation. Leg measurements: I 3.35 (1.02, 1.14, 0.76, 0.43); II 3.22 (1.02, 1.08, 0.72, 0.40); III 2.56 (0.79, 0.79, 0.60, 0.38); IV 2.70 (0.83, 0.87, 0.62, 0.38).

**Figure 9.**
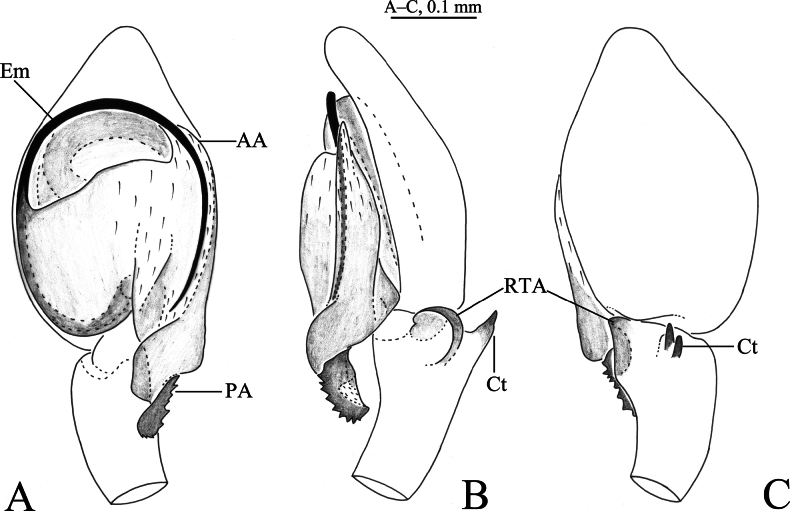
*Sudesna medog* sp. nov. holotype male (**A–C**). **A**. Left male palp, ventral view; **B**. Same, retrolateral view; **C**. Same, dorsal view.

**Figure 10.**
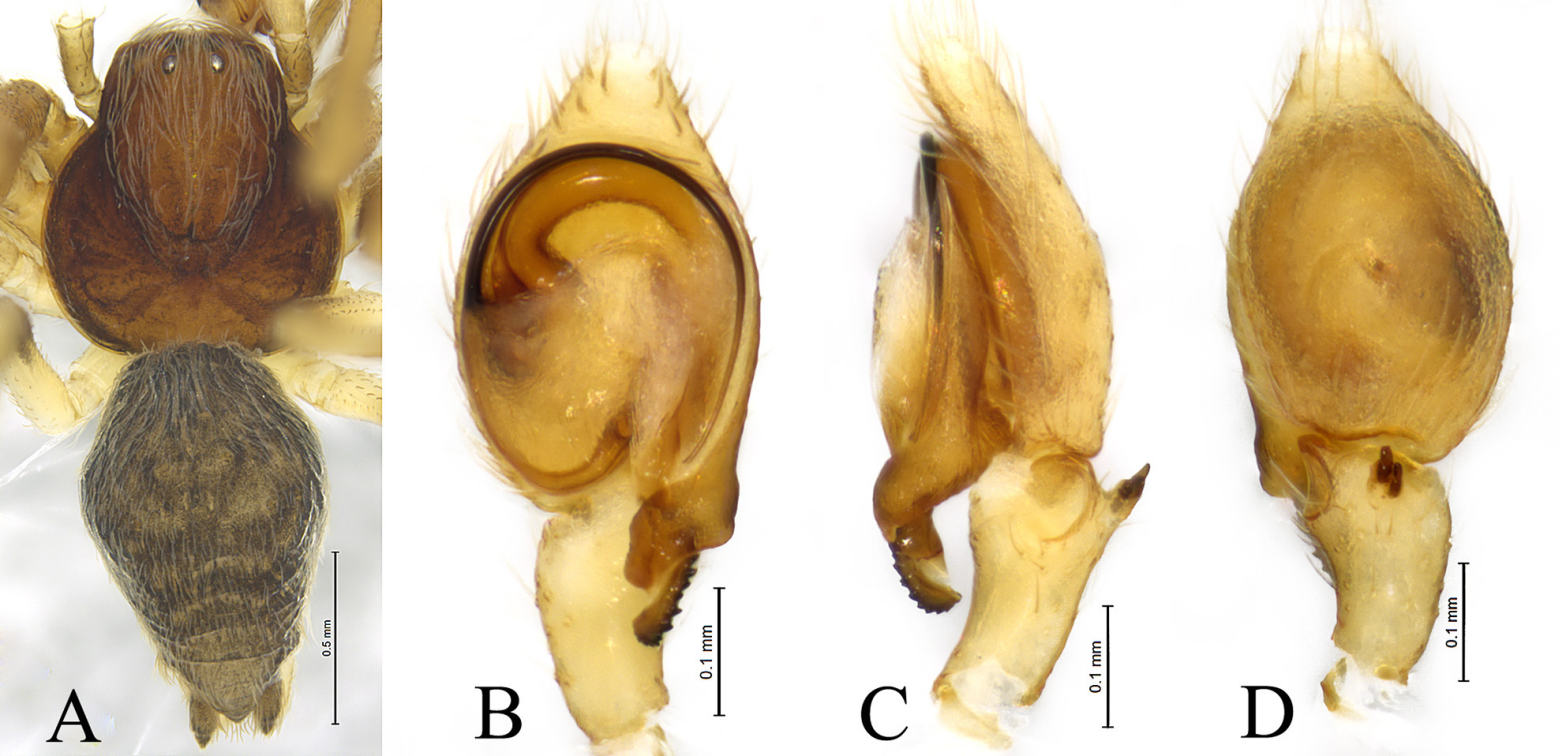
*Sudesna medog* sp. nov. holotype male (**A–D**). **A**. Male habitus, dorsal view; **B**. Left male palp, ventral view; **C**. Same, retrolateral view; **D**. Same, dorsal view.

***Palp*** (Figs 9A–C, 10B–D). Tibia with 2 ctenidia dorsally, almost located at the distal-most part of tibia. Retrolateral tibial apophysis hood-shaped in retrolateral view and triangular in dorsal view. Embolus semicircular and originating at about 9:00 o’clock position and terminating at about 5:00 o’clock position. Anterior arm of conductor (AA) short and membranous; posterior arm (PA) spiral, with serrated tip.

**Female** unknown.

##### Distribution.

Known only from the type locality, Xizang, China (Fig. 13).

#### Sudesna wangi sp. nov.

Taxon classificationAnimaliaAraneaeDictynidae

E2E0A5BD-7DA4-552A-8612-10CCA37649A3

https://zoobank.org/5A035036-43BB-4B06-8F67-613B3C86A1BA

Figs 11, 12, 13

##### Chinese name.

王氏苏蛛.

##### Type material.

***Holotype***: ♂ (SWUC-T-DI-23-01) • China, Xizang, Nyingchi City, Chayu County, Cibagou National Nature Reserve, 28°36'01"N, 97°04'00", elev. 1995 m, 22.06.2023, C. Wang leg. ***Paratypes***: • 1 ♂ 1 ♀ (SWUC-T-DI-21-02 to 03), same data as holotype.

##### Etymology.

This species is named after the collector of the type material; a noun in genitive case.

##### Diagnosis.

**Figure 11.**
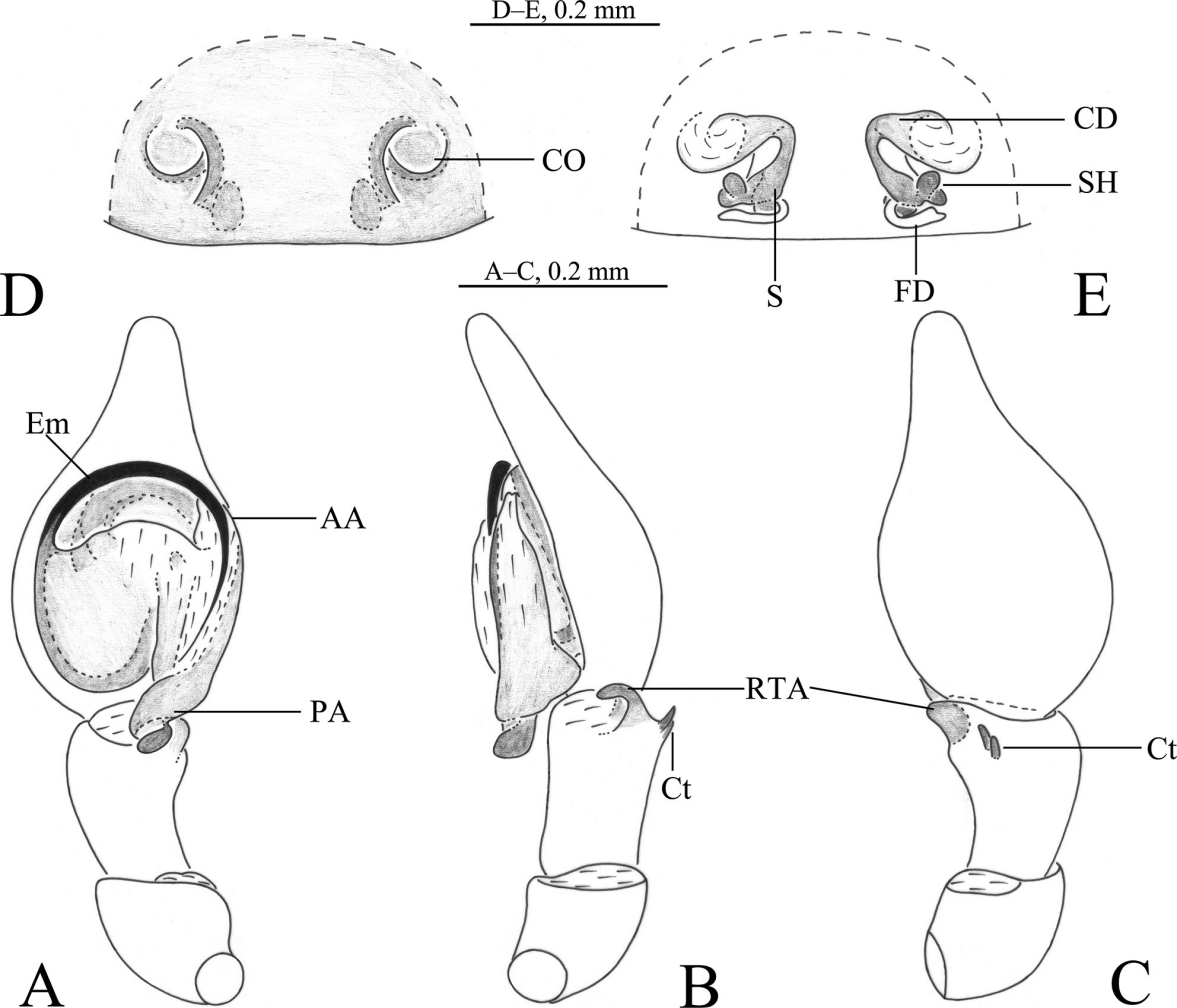
*Sudesna wangi* sp. nov. holotype male (**A–C**) and paratype female (**D, E**). **A**. Left male palp, ventral view; **B**. Same, retrolateral view; **C**. Same, dorsal view; **D**. Epigyne, ventral view; **E**. Vulva, dorsal view. Abbreviations.

**Figure 12.**
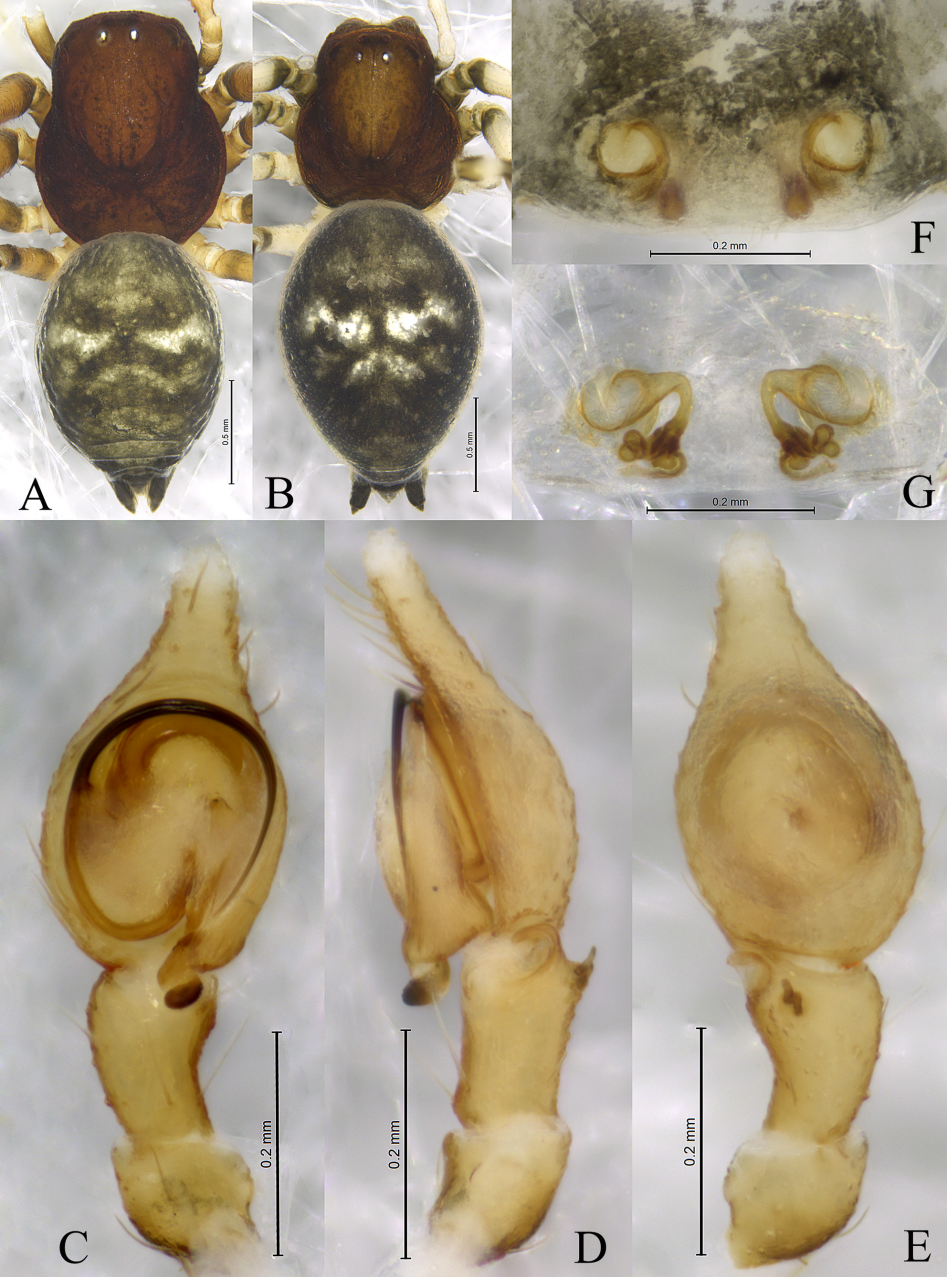
*Sudesna wangi* sp. nov. holotype male (**A, C–E**) and paratype female (**B, F, G**). **A**. Male habitus, dorsal view; **B**. Female habitus, dorsal view; **C**. Left male palp, ventral view; **D**. Same, retrolateral view; **E**. Same, dorsal view; **F**. Epigyne, ventral view; **G**. Vulva, dorsal view.

The male of this new species is similar to *S. flavipes* (Hu, 2001) (Figs 3A–C, 4C–E) in having a somewhat O-shaped embolus, and spiral, with a rounded end posterior arm (PA) of the conductor, but it can be distinguished by the originating 10:00 o’clock position of the embolus (vs 9:00), the robust posterior arm of the conductor (vs slender) (Figs 11A, 11B, 12C, 12D). The female of this new species is similar to *S. yangi* Wang, Peng & Zhang, 2025 (Wang et al. 2025: 144, figs 13D, E, 14F, G) in having large, 9-shaped copulatory openings, and long, abruptly bent copulatory ducts, but it can be distinguished by the globular spermatheca (vs clavate), and the pair of spermathecal heads (vs only one) (Figs 11E, 12G).

##### Description.

**Male holotype** (Fig. 12A) total length 2.33. Prosoma 1.17 long, 0.92 wide; Opisthosoma 1.26 long, 0.93 wide. Eye sizes and interdistances: AME 0.05, ALE 0.06, PME 0.05, PLE, 0.05; AME–AME 0.08, AME–ALE 0.13, PME–PME 0.11, PME–PLE 0.18, ALE–PLE 0.02. MOA 0.15 long, anterior width 0.19, posterior width 0.20. Clypeus height 0.17. Chelicerae stout, brown, with 4 promarginal teeth and 2 retromarginal teeth. Legs yellowish, with black pigmentation. Leg measurements: I 3.33 (1.00, 1.10, 0.77, 0.46); II 3.24 (1.03, 1.03, 0.72, 0.46); III 2.57 (0.77, 0.81, 0.62, 0.37); IV 2.78 (0.89, 0.84, 0.68, 0.37).

***Palp*** (Figs 11A–C, 12C–E). Tibia with 2 ctenidia dorsally, almost located at the distal-most part of tibia. Retrolateral tibial apophysis hook-shaped in retrolateral view and triangular in dorsal view. Embolus somewhat O-shaped, originating at about 10:00 o’clock position and terminating at about 5:00 o’clock position. Anterior arm of conductor (AA) short and membranous; posterior arm (PA) spiral, with a rounded end.

**Female paratype** (SWUC-T-DI-23-02, Fig. 12B) total length 2.50. Prosoma 1.00 long, 0.91 wide; opisthosoma 1.58 long, 1.09 wide. Eye sizes and interdistances: AME 0.04, ALE 0.05, PME 0.05, PLE, 0.05; AME–AME 0.08, AME–ALE 0.10, PME–PME 0.11, PME–PLE 0.15, ALE–PLE 0.03. MOA 0.16 long, anterior width 0.17, posterior width 0.18. Clypeus height 0.14. Legs yellowish, with black pigmentation. Leg measurements: I 3.24 (0.99, 1.09, 0.72, 0.44); II 3.12 (0.97, 1.02, 0.70, 0.43); III 2.58 (0.83, 0.83, 0.59, 0.33); IV 3.05 (1.01, 0.98, 0.70, 0.36).

***Epigyne*** (Figs 11D, 11E, 12F, 12G). Copulatory openings large, 9-shaped, facing away from each other, separated about 3 times their width. Copulatory ducts slightly sclerotized, long, abruptly bent. Spermatheca almost globular, spermathecal heads somewhat ball-shaped. Fertilization ducts length about 2.5 times the width of copulatory openings, curved as semicircular, directed laterally.

##### Variation.

Male (*N* = 2) total length 2.17–2.33.

##### Distribution.

**Figure 13.**
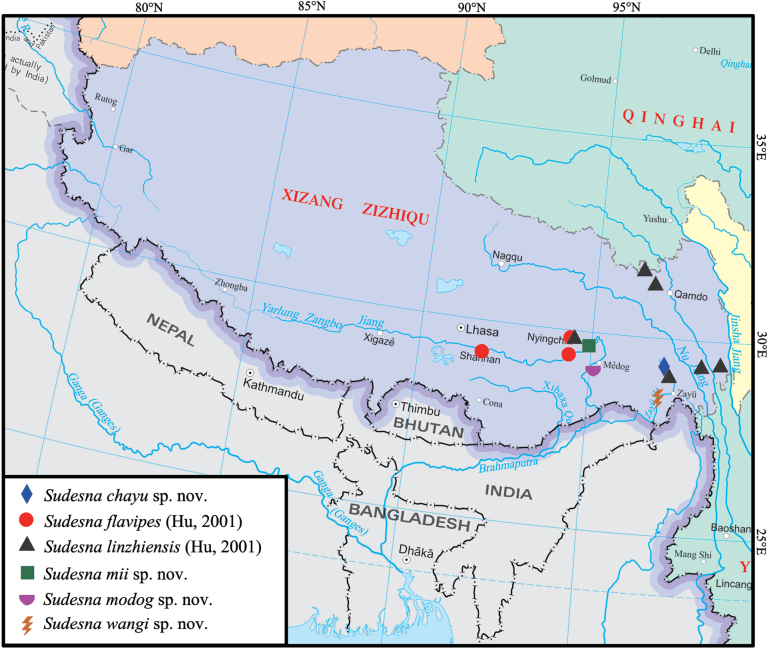
Distribution of *Sudesna* species in Xizang, China.

Known only from the type locality, Xizang, China (Fig. 13).

## Supplementary Material

XML Treatment for Sudesna

XML Treatment for Sudesna chayu

XML Treatment for Sudesna flavipes

XML Treatment for Sudesna linzhiensis

XML Treatment for Sudesna mii

XML Treatment for Sudesna medog

XML Treatment for Sudesna wangi

## Additional information

### Artificial Intelligence (AI) use

The authors accept full responsibility for the content of the manuscript, including the disclosure of any use of AI.

No AI tools were used in the preparation of this manuscript.

### Author ORCIDs

L.-Y. Wang https://orcid.org/0000-0002-5250-3473

Z.-S. Zhang https://orcid.org/0000-0002-9304-1789

Y.-Q. Xu https://orcid.org/0000-0002-1889-9531

### Data availability

All of the data that support the findings of this study are available in the main text.
